# METTL3 promotes colorectal cancer metastasis by promoting the maturation of pri-microRNA-196b

**DOI:** 10.1007/s00432-022-04429-9

**Published:** 2022-11-08

**Authors:** Lanlan Huang, Danlu Liang, Yu Zhang, Xiaoting Chen, Junxiong Chen, Chuangyu Wen, Huanliang Liu, Xiaorong Yang, Xiangling Yang, Shaoqiang Lin

**Affiliations:** 1 Department of Clinical Laboratory, The First Affiliated Hospital of Guangdong Pharmaceutical University, Guangzhou, China; 2 School of Clinical Medicine, Guangdong Pharmaceutical University, Guangzhou, China; 3 Guangdong Provincial Key Laboratory of Colorectal and Pelvic Floor Diseases, Guangdong Institute of Gastroenterology, The Sixth Affiliated Hospital, Sun Yat-Sen University, Guangzhou, China; 4 Department of Clinical Laboratory, The Sixth Affiliated Hospital, Sun Yat-Sen University, Guangzhou, China

**Keywords:** METTL3, m6A, miR-196b, Cancer metastasis, Colorectal cancer

## Abstract

**Purpose:**

Methyltransferase-like 3 (METTL3), a key member of the m6A methyltransferase complex, is upregulated in multiple human malignancies and plays a role in regulating tumor migration. This study aimed to reveal the underlying mechanism by which METTL3 in regulates the metastasis of colorectal cancer (CRC).

**Methods:**

We compared METTL3 expression levels in CRC tumor tissues and adjacent nontumor tissues by immunohistochemistry (IHC). The functional roles of METTL3 in CRC were assessed by real-time cell migration assays, wound-healing assays and Transwell assays. miRNA sequencing (miRNA-seq), RNA-binding protein immunoprecipitation (RIP) assays and N6-methyladenosine immunoprecipitation (MeRIP) assays were performed to confirm the molecular mechanism underlying the involvement of METTL3 in CRC cell metastasis.

**Results:**

We found that METTL3 was overexpressed in CRC tissues. METTL3 knockdown significantly inhibited CRC cell migration and invasion, while METTL3 overexpression had the opposite effects. Furthermore, we demonstrated that METTL3 regulates miR-196b expression via an N6-methyladenosine (m6A)-pri-miR-196b-dependent mechanism and thereby promotes CRC metastasis.

**Conclusion:**

This study shows the important role of METTL3 in CRC metastasis and provides novel insight into m6A modification in CRC metastasis.

**Supplementary Information:**

The online version contains supplementary material available at 10.1007/s00432-022-04429-9.

## Introduction

Colorectal cancer (CRC) is one of the most common malignant tumors among both men and women (Sung et al. 2021; Siegel et al. 2021). Due to improvements in detection and treatment, the 5-year relative survival rate of CRC has dramatically increased (Siegel et al. 2021). Patients diagnosed early with only localized disease have a 5-year survival rate of 90%. Nevertheless, this rate drops to 72% and 14% for patients diagnosed with regional and distant-stage disease, respectively (Siegel et al. 2021). Therefore, identifying the factors involved in CRC tumorigenesis and progression is imperative to find novel potential targets for improving the clinical outcomes of patients with metastatic CRC.

N6-methyladenosine (m6A) is the most abundant chemical modification in eukaryotic messenger RNAs (mRNAs) (Meyer et al. 2012) and preferentially occurs in the consensus motif “RRACH” (R=G or A; H=A, C, or U) (Dominissini et al. 2012; Meyer et al. 2012; Bodi et al. 2010). Recent studies have shown that m6A modification may have a profound impact on multiple aspects of RNA metabolism, including the nuclear export, splicing, stability and translation of target mRNAs (Chen et al. 2020b; Kasowitz et al. 2018; Du et al. 2016; Wang et al. 2014, 2015; Roundtree et al. 2017). m6A modification has been shown to affect cell meiosis, the circadian clock and stem cell self-renewal and differentiation (Weng et al. 2018; Xu et al. 2017; Zhong et al. 2018). RNA m6A modification is dynamically regulated and involves methylation by “writers”, recognition by “readers”, and demethylation by “erasers” (Liu et al. 2014; Jia et al. 2011; Sibbritt et al. 2013). Accumulating evidence indicates that the dysregulation of m6A regulatory enzymes is associated with cancer progression (Chen et al. 2018; Visvanathan et al. 2018; Zhu et al. 2020; Hua et al. 2018). Methyltransferase-like 3 (METTL3) is an m6A methylase that functions in methylation processes (Sibbritt et al. 2013), and the dysregulation of METTL3 expression has been reported in multiple human malignancies (Cheng et al. 2019; Chen et al. 2021b; Wang et al. 2020; Hua et al. 2018; Zhou et al. 2021a). An increasing number of studies have shown that METTL3 promotes cancer cell metastasis (Chen et al. 2021b, 2018; Cheng et al. 2019; Hua et al. 2018; Wang et al. 2020; Zhou et al. 2021a). In CRC, METTL3 has been reported to regulate metastasis by enhancing the mRNA stability of SOX2, HK2, GLUT1 and YPEL5 through an m6A-IGF2BP2/3-dependent mechanism (Li et al. 2019; Zhou et al. 2021a; Shen et al. 2020). However, the underlying mechanism of METTL3 in the metastasis of CRC remains largely unclear.

miRNAs are short, noncoding RNAs that play critical roles in diverse biological processes, including proliferation, apoptosis and metastasis (Wang et al. 2017; Kim et al. 2014; Meng et al. 2013; Ling et al. 2016; Andriani et al. 2018; El Bezawy et al. 2017). Mature miRNAs are generated via a two-step processing pathway to yield 19–24 nucleotide small RNAs that regulate gene expression at the posttranscriptional level (Lee et al. 2002). The first step is precise cleavage of the stem loops embedded in the primary transcripts (pri-miRNAs) by the microprocessor complex, composed of the RNA-binding protein DGCR8 and the type III RNase DROSHA, to release pre-miRNAs (Denli et al. 2004; Gregory et al. 2004; Han et al. 2004). Then, the pre-miRNAs are exported to the cytoplasm and diced by Dicer to generate ~ 22 nt miRNA duplexes (Hutvagner et al. 2001; Bohnsack et al. 2004). pri-miRNA processing is a critical step in miRNA biogenesis. This initial event requires recognition of the junction between the stem and the flanking single-stranded RNA of the pri-miRNA hairpin by DGCR8 (Han et al. 2006). m6A methyltransferases (METTL3, METTL14), an m6A-binding protein (HNRNPA2B1) and a demethylase (FTO) have been reported to affect miRNA expression levels (Berulava et al. 2015; Alarcon et al. 2015a, b; Han et al. 2019; Wang et al. 2019; Bi et al. 2021; Peng et al. 2019; Ma et al. 2017; Chen et al. 2020a; Zhou et al. 2021b). In recent years, an increasing number of researchers have demonstrated that METTL3 promotes cancer cell proliferation, apoptosis and metastasis by accelerating miRNA maturation in an m6A-dependent manner (Han et al. 2019; Wang et al. 2019; Bi et al. 2021; Peng et al. 2019).

In this study, we showed that METTL3 is upregulated in CRC and promotes CRC cell migration and invasion. Moreover, we first demonstrated that METTL3 can methylate pri-miR-196b, and then upregulate the expression of miR-196b, thereby increasing CRC cell migration ability.

## Materials and methods

### Clinical specimens

The CRC tissue microarray (TMA) slides used for immunohistochemistry (IHC) of METTL3 protein expression were purchased from Shanghai Outdo Biotech (Shanghai, China). The colon cancer TMA (HColA180Su09) contains 69 tumor tissues and 55 adjacent tissues. The rectal cancer TMA (HRec-Ade180Sur-04) contains 68 tumor tissues and 57 adjacent tissues. The CRC TMA slides used for in situ hybridization (ISH) analysis of miR-196b expression were obtained from the tumor bank of the Department of Pathology of the First Affiliated Hospital, Sun Yat-sen University (Guangzhou, China). The human sample collection procedure was approved by the Ethics Committee of the Sixth Affiliated Hospital, Sun Yat-sen University (Guangzhou, China), and written informed consent was obtained from all of the patients. All experimental protocols were carried out in accordance with the approved guidelines and were approved by the Ethics Committee of the Sixth Affiliated Hospital, Sun Yat-sen University (Guangzhou, China).

### Cell culture

The human CRC cell lines HCT 116, SW480 and Caco2 were purchased from the American Type Culture Collection (Manassas, VA, USA). Cells were cultured in RPMI 1640 medium or DMEM supplemented with 10% fetal bovine serum (vol/vol) and 1% penicillin‒streptomycin (Gibco, Grand Island, NY, USA) at 37 °C in 5% CO_2_.

### Lentiviral packaging and cell transduction

For lentiviral packaging and cell transduction, METTL3 knockdown or overexpression lentiviruses were obtained from Shanghai GeneChem Co., Ltd, China. For overexpression, cDNA was amplified by quantitative real-time PCR (qRT-PCR) and subcloned into the GV341 vector according to the manufacturer’s instructions. For stable silencing, shRNA lentiviruses (shMETTL3 with the target sequence GCCTTAACATTGCCCACTGAT) were constructed using GV248 vectors. For miR-196b, miR-196b precursor sequences were cloned into the GV309 vector. CRC cells at 20–30% confluence were plated in 24-well dishes and infected with METTL3 overexpression lentivirus (oeMETTL3), METTL3 knockdown lentivirus (shMETTL3), miR-196b overexpression lentivirus or the corresponding negative controls, respectively. Pools of stably transduced cells were generated by selection using puromycin (1 mg/ml) for 2 weeks. siMETTL3, miR-196b mimic and miR-196b inhibitor were ordered from RiboBio Co., Ltd, China (METTL3_1 with the target sequence CAAGTATGTTCACTATGAA; METTL3_2 with the target sequence GACTGCTCTTTCCTTAATA). Transfection was achieved using Lipofectamine 3000 (Invitrogen, Carlsbad, CA, USA) following the manufacturer’s protocols. After transfection, the expression of METTL3 and miR-196b was validated by qRT-PCR or Western blotting (WB).

### RNA extraction and qRT-PCR

Total RNA was extracted from cells using RNAiso Plus (Invitrogen, USA) according to the manufacturer’s protocol. RNA was reverse-transcribed to cDNA using the PrimeScript^TM^RT Reagent Kit and random primers (Takara, Dalian, China). qRT-PCR to assess pri-miR-196b expression was performed using SYBR Premix Ex Taq™ II (Takara), and expression data were normalized to GAPDH mRNA expression according to the manufacturer's instructions. The expression of mature miR-196b was analyzed using the All-in-One miRNA qRT-PCR Detection Kit (GeneCopoeia, Rockville, MD, USA). The small endogenous nucleolar RNU6B was used as a control for miRNA normalization. All experiments were performed in triplicate. Real-time PCR was performed using the Applied Biosystems 7900 Real-Time PCR System (Applied Biosystems, Foster City, CA, USA). Gene expression △Ct values from each sample were calculated by normalizing to an internal control (RNU6B/GAPDH), and relative expression was calculated using the formula 2^−△△Ct^ values. The primers used in this study were as follows: pri-miR-196b_F: CACCAGAACTGGTCGGTGATT and pri-miR-196b_R: TAATGAAGGCAGTGTCGTGCT; GAPDH_F: AGCCTCAAGATCATCAGC and GAPDH_R: GAGTCCTTCCACGATACC.

### WB

Cells were collected from cultured dishes and lysed in RIPA lysis buffer (Cell Signaling Technology, Danvers, MA, USA) supplemented with protease inhibitors. Briefly, equal amounts of protein were separated by 10% sodium dodecyl sulfate-polyacrylamide gel electrophoresis (SDS-PAGE) and transferred to a polyvinylidene fluoride (PVDF) membrane (Millipore, Billerica, MA, USA). The membranes were blocked with 5% skim milk for 1 h at room temperature and then incubated with primary antibodies overnight at 4 °C. The next day, after incubation with horseradish peroxidase-conjugated secondary antibodies for 1 h at room temperature, the signals were detected using a chemiluminescence ECL detection kit (Santa Cruz Biotechnology). Anti-METTL3 (1:1000 dilution, Abcam, Cambridge, UK) and anti-GAPDH (1:1000 dilution, Abcam, Cambridge, UK) antibodies were used.

### IHC

IHC staining was performed as described previously (Huang et al. 2018; Chen et al. 2021b). Briefly, TMA slides were incubated in a dry oven at 60 °C for 2 h. After deparaffinization and rehydration, antigen retrieval was performed by boiling the sections in a 0.1 mol/L citrate acid solution (pH = 6.0). Endogenous peroxidase activity was blocked using 0.3% H_2_O_2_ for 10 min at room temperature. The TMA slides were blocked with 5% normal goat serum (BOSTER, China) for 30 min and subsequently incubated with an anti-METTL3 antibody (1:1000, Abcam, Cambridge, UK) overnight at 4 °C. A staining index (with values ranging from 0 to 12) was calculated as follows the staining intensity (0, negative staining; 1, weak staining; 2, moderate staining; and 3, strong staining) multiplied by the proportion of positively stained tumor cells (0, < 5%; 1, 5–25%; 2, 25–50%; 3, 50–75%; and 4, ≥ 75%). The median value of the total staining score was 6; thus, a score of 0–6 indicated low expression, and a score of 8–12 indicated high expression.

### ISH

ISH was performed using miRNA-196b probe from Exiqon (Exiqon A/S, Denmark). The probe was detected using anti-digoxigenin-AP (Roche, Denmark), and the hybridized probes were detected by applying the BCIP/NBT Alkaline Phosphatase Color Development Kit. No-probe controls were included for both hybridization procedures. Images were taken with a Leica DMI 4000B inverted microscope (Leica Microsystems, Wetzlar, Germany). ISH staining of the images was analyzed using Image Pro-Plus (version 5.0, Media Cybernetics, Silver Spring, MD, USA) (Xavier et al. 2005; Huang et al. 2018).

### Real-time cell migration assays

Real-time cell migration assays were performed on the xCELLigence system from ACEA Biosciences. Briefly, the lower chamber of the CIM plate was filled with serum-containing medium, and the upper chamber was filled with serum-free medium. The cells were resuspended in serum-free medium, counted and seeded into the upper chamber by applying 5 × 10^4^ cells in 100 µL of medium. After addition of the cells, the CIM plate was incubated for 30 min at room temperature and then blocked in an RTCA DP instrument. The CI values were measured automatically every 15 min for 3 days.

### Wound healing assays

Wound healing assays were performed with Ibidi Culture-Insert 2 Well. Briefly, 2 × 10^5^ cells were seeded into each well of the Culture-Insert 2 Well. The Culture-Insert 2 Well was removed after 24 h, and the cell layer was washed with PBS to remove cell debris and nonattached cells. The dish was filled with medium, and the wound-healing process was monitored under a microscope. Each assay was repeated 3 times.

### Migration assays

Cell migration assays were carried out with transwell chambers (BD Bioscience, San Jose, CA, USA). HCT 116 cells (5 × 10^4^) or SW480 cells (8 × 10^4^) were seeded in the upper chamber in serum-free medium, and the lower chamber was filled with medium containing 10% FBS. After incubation at 37 °C in 5% CO_2_ for a suitable time (HCT 116 cells: 27 h, SW480 cells: 26 h), cells in the upper chambers were fixed with 4% paraformaldehyde for 15 min, stained with 0.1% crystal violet and photographed using a microscope. All studies were repeated at least three times in triplicate.

### Invasion assays

Invasion assays were performed as described previously (Huang et al. 2018). Briefly, Transwell chambers precoated with Matrigel (BD Bioscience, San Jose, CA, USA) were used to perform the invasion assay. A total of 1 × 10^5^ cells were cultured in serum-free medium in the upper chambers of each well, while medium containing 10% FBS was added to the lower chamber of the well. After 30 h, the cells were fixed with 4% polyoxymethylene and stained with crystal violet. Cells on the upper side of the membrane that had not migrated were gently wiped off, and the stained cells on the lower side were observed under a microscope. The number of migrated cells in five fields per chamber was counted, and the average values were calculated.

### RIP assays

After formaldehyde-crosslinking (0.3% for 10 min), we isolated the nuclear fraction from 4 × 10^7^ cells using NE-PER Nuclear and Cytoplasmic Extraction Reagents (Thermo Scientific, Rockford, IL, USA). The nuclear fraction was lysed in RIP Lysis Buffer (Millipore). RIP was performed using the Magna RIP RNA-Binding Protein Immunoprecipitation Kit (Millipore) following the manufacturer’s protocol. For endogenous immunoprecipitation we used 5 μg of an anti-METTL3 rabbit antibody (anti-METTL3-1#: catalog No. A301-567A; anti-METTL3-2#: catalog No. A301-568A. Bethyl, Montgomery, UK) or normal rabbit IgG as a control bound to protein A magnetic beads (Millipore). After immunoprecipitation, each immunoprecipitate was resuspend in 150 μL of proteinase K buffer to digest the proteins, and then the supernatant was transferred to a new tube. A total of 250 μL of RIP wash buffer and 400 μL of phenol:chloroform:isoamyl alcohol were added to each tube. The tubes were vortexed for 15 s and centrifuged at 14,000 rpm for 10 min. Then, the aqueous solution was precipitated and resuspended in 10 μL of RNase-free water. The RNA was analyzed by qRT-PCR.

### MeRIP assays

The nuclear fraction from 4 × 10^7^ cells was isolated using NE-PER Nuclear and Cytoplasmic Extraction Reagents (Thermo Scientific). RNA was extracted from the nuclear fraction using TRIzol (Invitrogen) and then fragmented into sizes between 60 and 200 nucleotides in length using Ambion^®^ RNA Fragmentation Reagents (Thermo Scientific). The fragmented RNA was precipitated by adding one-tenth volumes of 3 M sodium acetate (pH 5.2) and glycogen (100 μg/mL final) and 2.5 volumes of 100% ethanol. Rabbit anti-m6A antibody (Synaptic Systems, Germany) and rabbit normal IgG control bound to protein A magnetic beads (Millipore) were used for immunoprecipitation. The immunoprecipitated RNA was extracted using phenol:chloroform:isoamyl alcohol. Then, the aqueous solution was precipitated and resuspended in 10 μL of RNase-free water. The RNA was analyzed by qRT-PCR.

### Statistical analysis

Statistical analysis was performed using SPSS18.0 software (SPSS, IBM, Chicago, IL, USA). The data are expressed as the mean ± SD, and statistical significance was determined with Student’s *t* tests. Statistical comparisons between groups were analyzed using Student’s paired *t* test. *P* values less than 0.05 were considered as statistically significant. The relationships between miR-196b expression and clinicopathological features were analyzed using the chi-square test.

## Results

### METTL3 is upregulated in human CRC

**Fig. 1:**
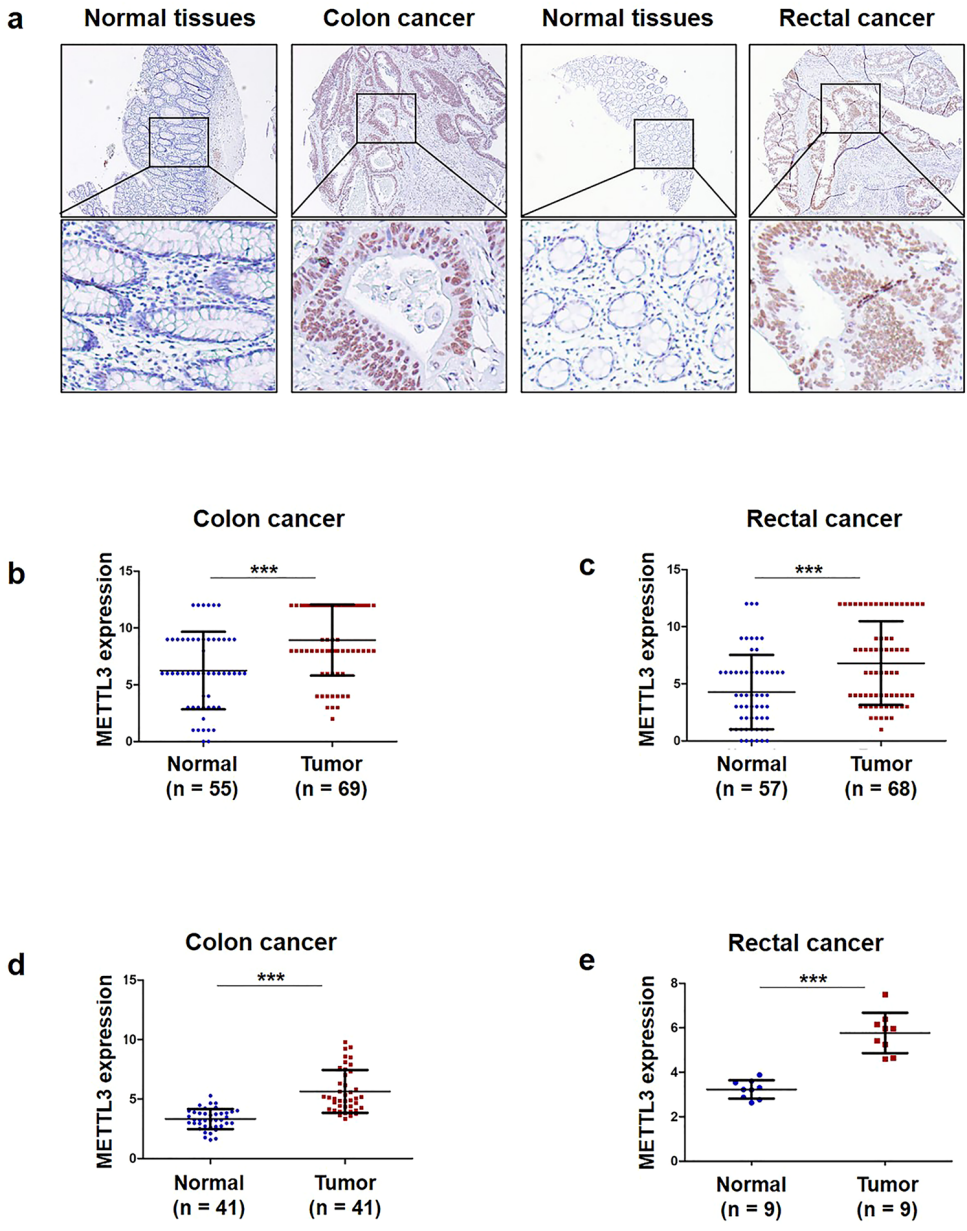
METTL3 is upregulated in CRC. **a** Representative image following IHC staining of CRC tumor tissues and adjacent normal tissues with anti-METTL3 antibody. **b** METTL3 protein expression in 69 colon cancer tissues and 55 adjacent normal tissues. Statistical significance was determined by two-tailed, unpaired Student’s *t* test. **c** METTL3 protein expression in 68 rectal cancer tissues and 57 adjacent normal tissues. Statistical significance was determined by two-tailed, unpaired Student’s *t* test. **d**–**e** METTL3 mRNA levels from 41 pairs of colon cancer tissues and 9 pairs of rectal cancer tissues from TCGA database. Statistical significance was determined by a two-tailed, paired Student’s *t* test. (***P < 0.001)

To better understand METTL3 protein expression and localization in CRC, we detected METTL3 expression in CRC tissues and adjacent tissues using IHC. We found that METTL3 expression was mainly localized in the nucleus (Fig. 1a). Furthermore, METTL3 was significantly upregulated in the CRC tissues (Fig. 1b, c). Next, we examined METTL3 expression in The Cancer Genome Atlas (TCGA) datasets (http://cancergenome.nih.gov). Consistent with our findings, METTL3 was significantly upregulated in CRC tissues compared with adjacent normal tissues (Fig. 1d, e).

### Decreased METTL3 expression inhibits CRC cell migration and invasion in vitro

**Fig. 2:**
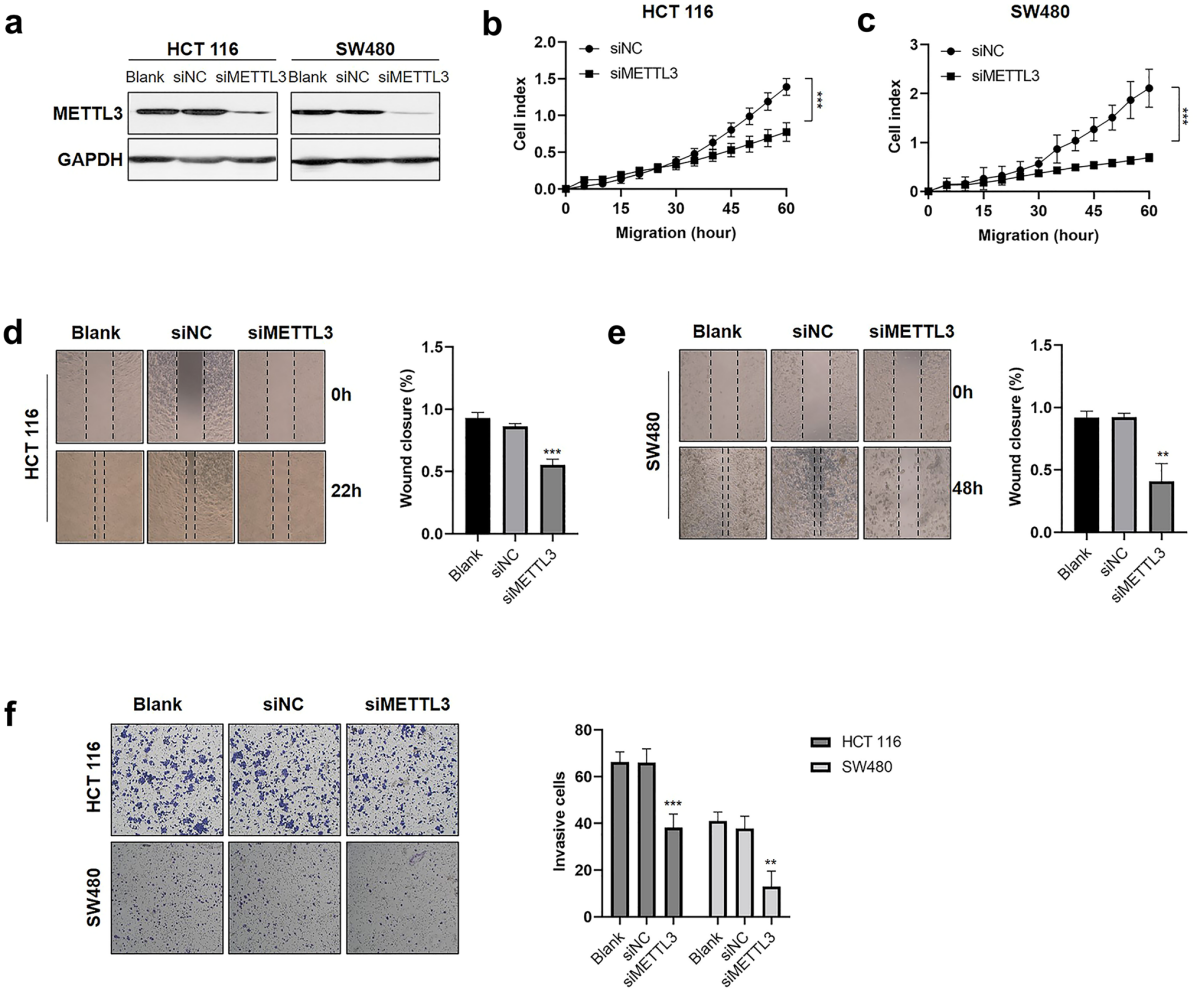
Knockdown of METTL3 inhibited CRC cell migration and invasion in vitro*.* **a** WB analysis of METTL3 expression in CRC cells infected with siNC or siMETTL3-1. **b**–**c** Real-time migration of CRC cells transfected with siNC or siMETTL3. The delta cell index indicates electrical impedance. **d**–**e** Wound healing assays were performed to investigate the effects of siMETTL3 on the migration of CRC cells. **f** Transwell invasion assays were performed to estimate the effects of siMETTL3 on CRC cell invasion. The data are presented as the mean ± SD. Three independent assays were performed (**d**–**e**) (**P < 0.01, ***P < 0.001; Student’s *t* test)

To investigate the role of METTL3 in CRC metastasis, we silenced METTL3 in HCT 116 and SW480 cells using METTL3 short hairpin RNAs (shRNAs) and siMETTL3_1. Knockdown of METTL3 expression was confirmed by WB and qRT-PCR (Fig. 2a, Fig. S1 and Fig. S2a, b). Real-time cell migration (Fig. 2b, c) and wound-healing (Fig. 2d, e and Fig. S2c, d) assays showed that the knockdown of METTL3 dramatically suppressed CRC cell migration. Consistently, knockdown of METTL3 markedly reduced cell invasion ability (Fig. 2f and Fig. S2e, f).

### Overexpression of METTL3 promotes cell migration and invasion in vitro

**Fig. 3:**
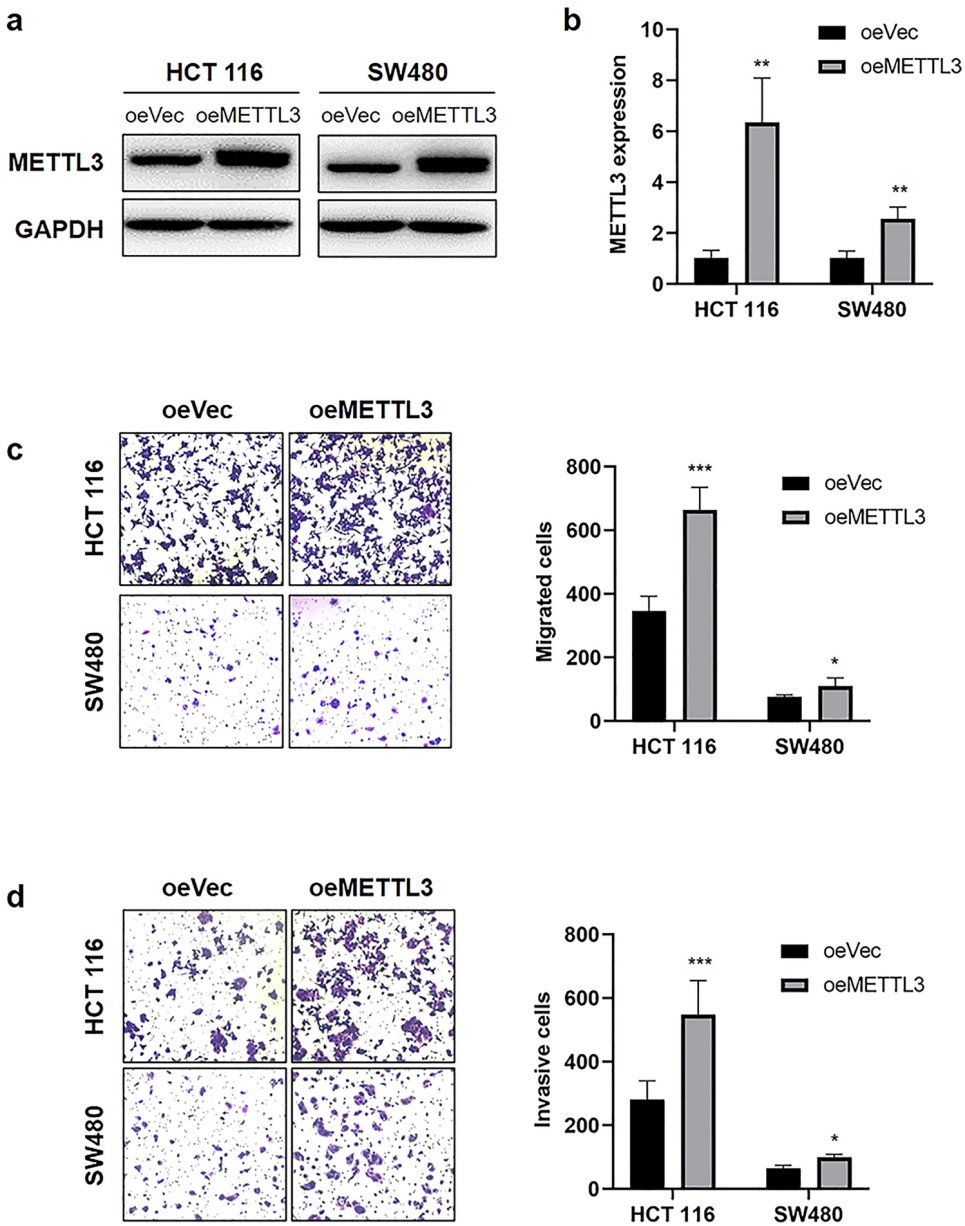
Overexpression of METTL3 promoted CRC cell migration and invasion in vitro. **a**–**b** WB and qRT-PCR analysis of METTL3 expression of in CRC cells infected with oeNC or oeMETTL3. **c** Migration assays revealed that overexpression of METTL3 increased the migration of HCT 116 and SW480 cells. **d** Transwell invasion assays revealed that overexpression of METTL3 increased the invasion of HCT 116 and SW480 cells. The data are presented as the mean ± SD. Three independent assays were performed (*P < 0.05, **P < 0.01, ***P < 0.001; Student’s *t* test)

We then transfected CRC cells with METTL3 lentiviral vectors. The expression of METTL3 was confirmed by WB and qRT-PCR (Fig. 3a, b). Transwell migration assays showed that METTL3 overexpression substantially increased the migration of CRC cells (Fig. 3c). In addition, Transwell invasion assays showed that METTL3 overexpression increased CRC cell invasion (Fig. 3d). Our findings indicated that METTL3 overexpression increases migration and invasion in CRC cells.

### Knockdown of METTL3 reshapes the miRNA profile of CRC cells

**Fig. 4:**
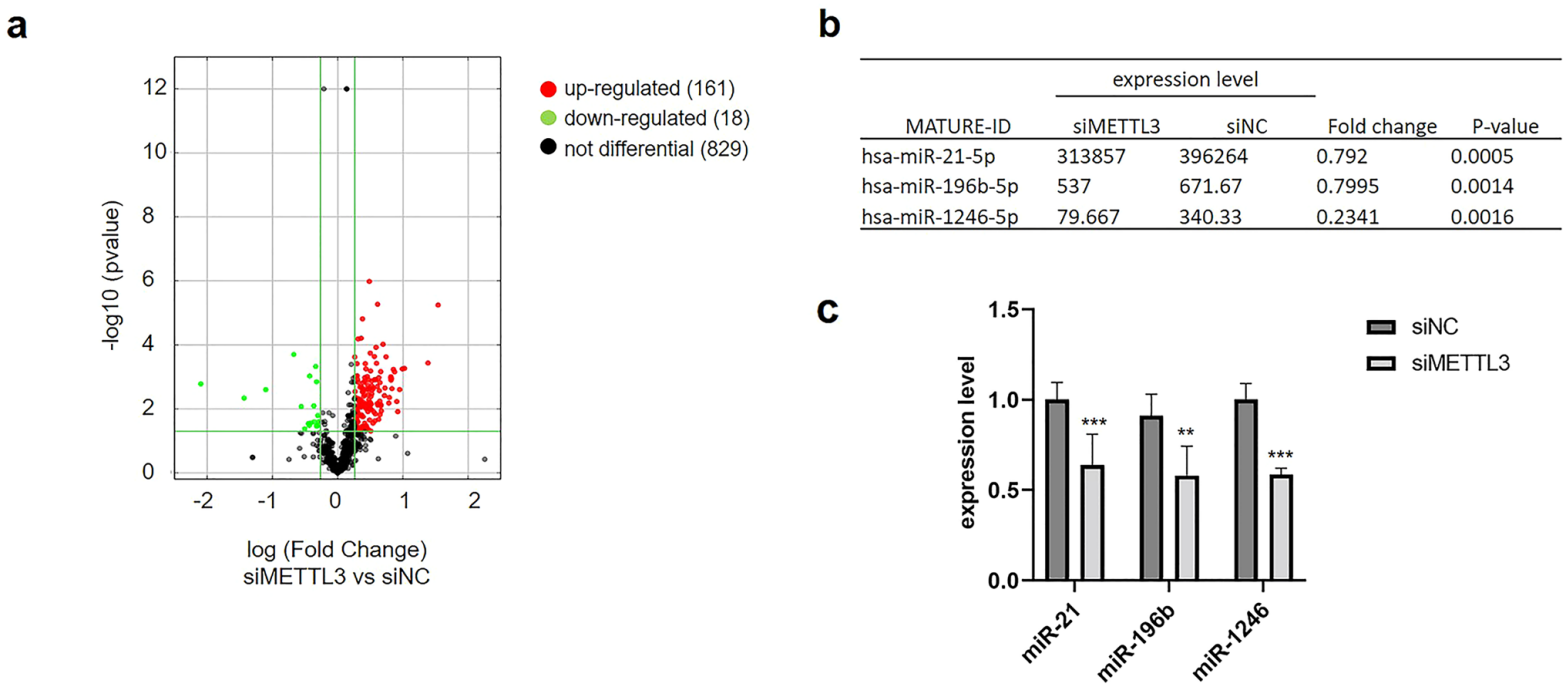
miRNA-seq revealed the miRNA profile of CRC cells. **a** The volcano plot shows the differential expression of miRNAs in HCT 116 cells treated with siMETTL3 detected with miRNA-seq. **b** The top 3 miRNAs are listed in the table. **c** qRT-PCR was used to measure the expression levels of the indicated miRNAs. The data are presented as the mean ± SD. Three independent assays were performed (**P < 0.01, ***P < 0.001; Student’s *t* test)

Since addition of the m6A mark acts as a key posttranscriptional modification that promotes the initiation of miRNA biogenesis, we next measured the global impact of METTL3 depletion on miRNA levels by performing miRNA-seq of RNA collected from control or METTL3 knockdown HCT116 cells. miRNA-seq revealed 179 miRNAs that were significantly differentially expressed (*P* < 0.05) by at least a 1.2-fold between the control and METTL3 knockdown cells. Of these miRNAs, 161 miRNAs were upregulated, and 18 miRNAs were downregulated (Fig. 4a and Table S1). We selected significantly downregulated miRNAs according to their fold-change, *P* value and miRNA abundance (Isoform > 300) (Fig. 4b). The expression of these miRNAs was verified by qRT-PCR, and the results showed that METTL3 knockdown significantly decreased the expression levels of miR-21-5p, miR-196b-5p, and miR-1246-5p (Fig. 4c). Previous studies have reported that m6A modification-dependent pri-miRNA processing is essential for the maturation of miR-21-5p (Liu et al. 2021; Wang et al. 2021) and miR-1246-5p (Huang et al. 2021; Peng et al. 2019), but its role in the maturation of miR-196b has not yet been investigated. Therefore, our study focused on whether METTL3 plays a role by regulating miR-196b in CRC cells.

### METTL3-dependent m6A methylation regulates the maturation of miR-196b

**Fig. 5:**
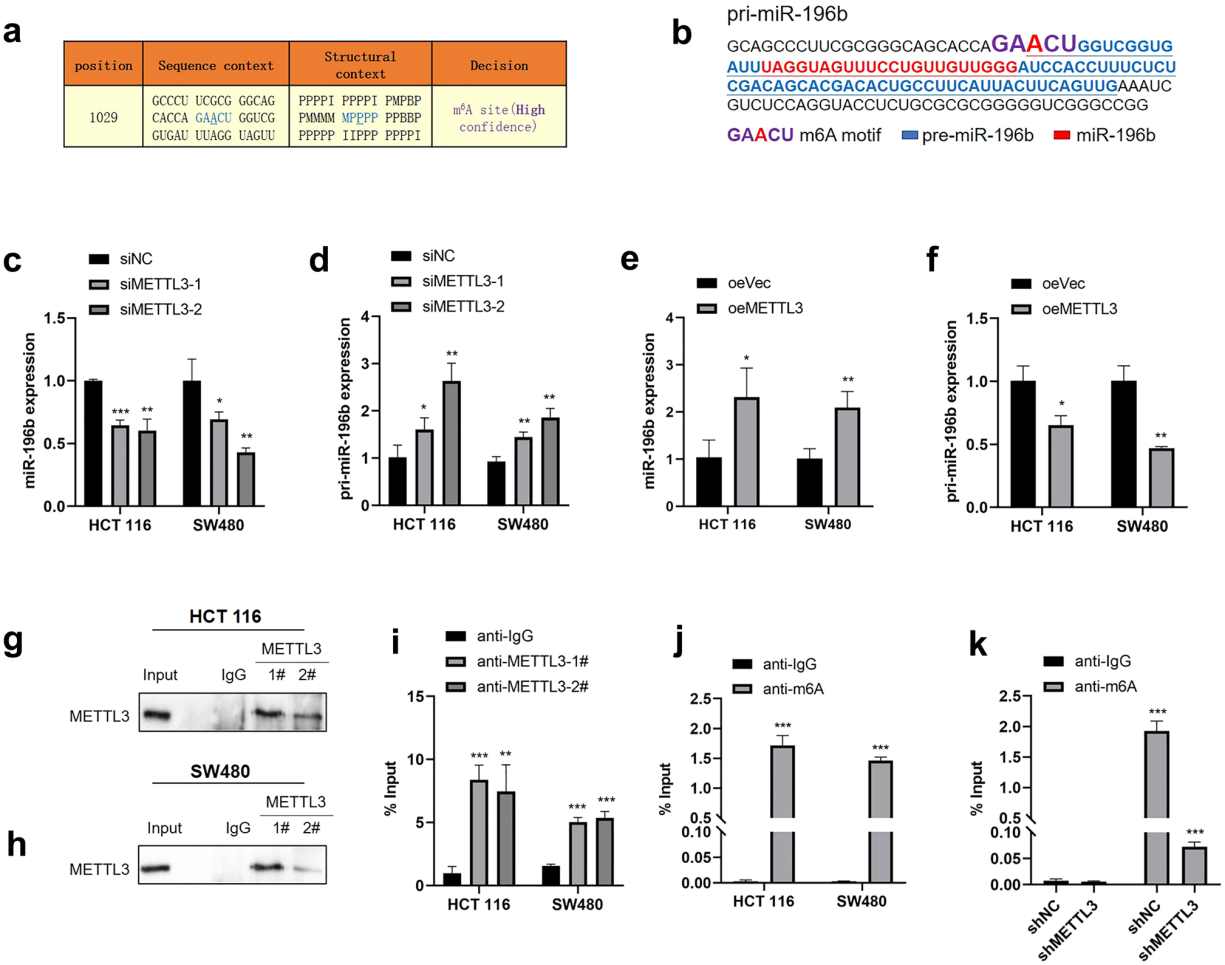
METTL3 regulates the processing of miR-196b. **a**–**b** Possible m6A methylation modification sites in miR-196b. **c**–**d** qRT-PCR analysis of miR-196b and pri-miR-196b level in CRC cells treated with siNC or siMETTL3. **e–f** qRT-PCR analysis of miR-196b and pri-miR-196b expression in CRC cells treated with oeNC or oeMETTL3. **g**–**h** Immunoprecipitation of endogenous METTL3. **i** pri-miRNAs bound to endogenous METTL3 were extracted and quantified by qRT-PCR. **j** m6A modification level of pri-miR-196b in CRC cells assessed by MeRIP. **k** m6A modification level of pri-miR-196b in SW480 cells treated with shMETTL3 detected by MeRIP. The data are presented as the mean ± SD. Three independent assays were performed (**c**–**f**) (*P < 0.05, **P < 0.01, ***P < 0.001; Student’s *t* test)

To further verify that METTL3 regulates miR-196b maturation by catalyzing m6A formation in RNAs, we obtained the full sequences of pri-miR-196b and pre-miR-196b from UCSC and miRBase. Then, we analyzed whether the single-stranded RNA flanking the pri-miR-196b contains the RRACH motif for m6A modification (R=G or A; H=A, C or U) (Dominissini et al. 2012; Meyer et al. 2012; Bodi et al. 2010). SRAMP (http://www.cuilab.cn/sramp/), which is a computational predictor of mammalian m6A sites (Zhou et al. 2016), revealed m6A motifs localized precisely at the pri-miR-196b splicing site (Fig. 5a, b). qRT-PCR primers were designed to amplify the putative m6A region of pri-miR-196b (Fig. S3). Knockdown of METTL3 expression was confirmed by WB and qRT-PCR (Fig. S1). qRT-PCR showed that the knockdown of METTL3 significantly decreased the expression of miR-196b (Fig. 5c) but increased the expression of pri-miR-196b in CRC cells (Fig. 5d), while the overexpression of METTL3 increased the expression of miR-196b (Fig. 5e) but reduced the expression of pri-miR-196b in CRC cells (Fig. 5f). Furthermore, miR-196b expression positively correlated with METTL3, DGCR8 and HNRNPA2B1 expression in CRC tissues (Fig. S4). To determine whether METTL3 binds the m6A methylation region of pri-miR-196b in vitro, we conducted RIP-qRT-PCR experiments using two antibodies that map to different regions of endogenous METTL3 (anti-METTL3-1#; anti-METTL3-2#). We incubated cells in formaldehyde for in vitro crosslinking, isolated the nuclear fractions and immunoprecipitated endogenous METTL3. After METTL3 immunoprecipitation, WB (Fig. 5g, h) and qRT-PCR (Fig. 5i) revealed that METTL3 interacts with the m6A methylation region of pri-miR-196b. To determine whether m6A modification was present in the pri-miR-196b regions, we conducted MeRIP-qRT-PCR by immunoprecipitating nuclear RNA from HCT 116 and SW480 cells with an anti-m6A antibody, followed by qRT-PCR. The results showed that m6A modification was enriched at the pri-miR-196b sequence (Fig. 5j). Moreover, the level of pri-miR-196b m6A modification was significantly reduced following METTL3 silencing in SW480 cells (Fig. 5k). Taken together, these results indicated that METTL3 regulates the processing of miR-196 in an m6A-dependent manner.

### miR-196b is required for the METTL3-induced enhancement of CRC cells migrations

**Fig. 6:**
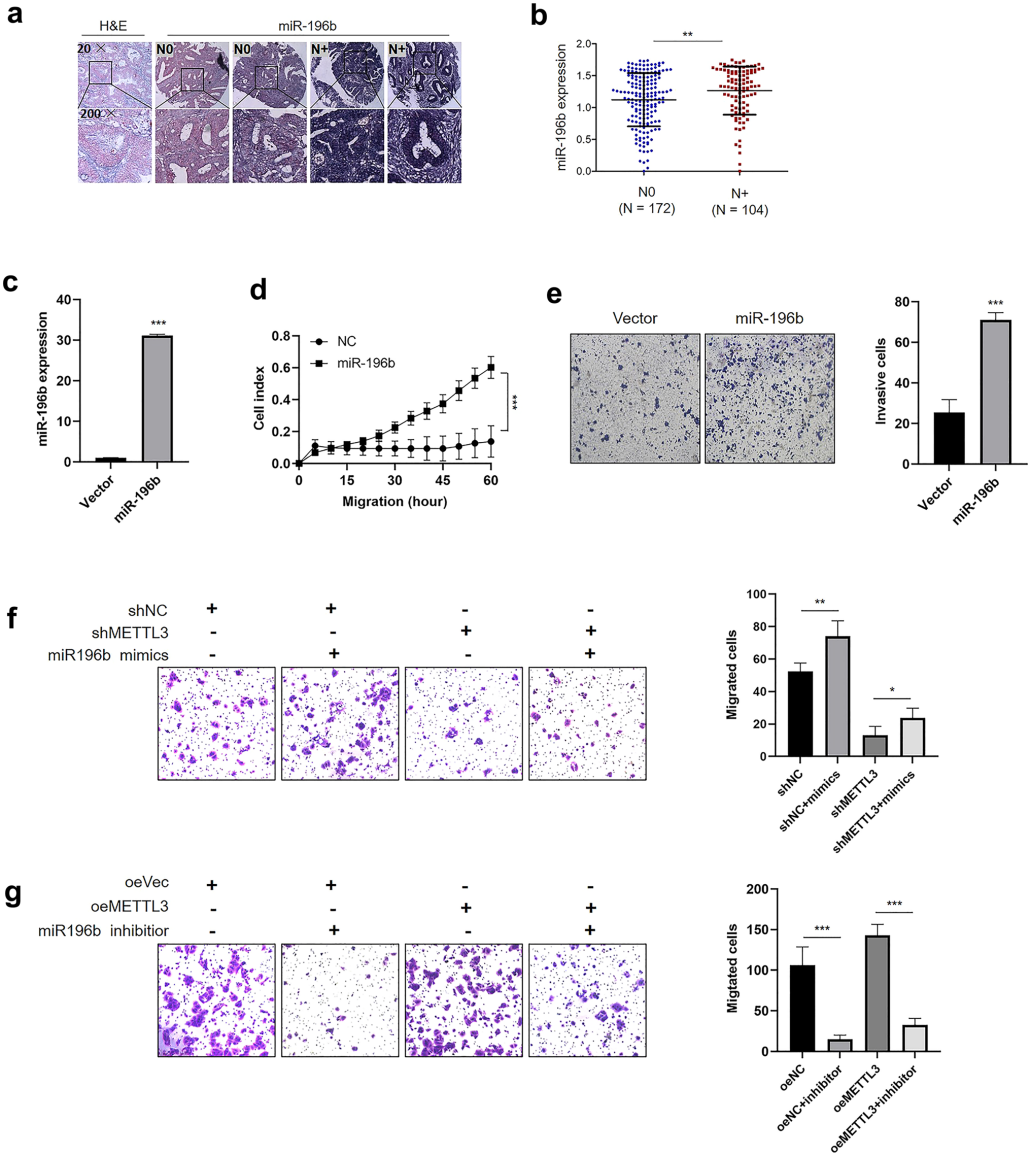
miR-196b rescued the METTL3-induced migration of CRC cells. **a** Representative ISH images showing miR-196b. **b** ISH analysis of paraffin blocks of CRC specimens was followed by analysis of miR-196b levels. miR-196b expression levels in patients with CRC with lymph node metastases were significantly higher than those in patients with CRC without lymph node metastasis. **c** qRT-PCR analysis of miR-196b expression in CRC cells infected with pLV-miR-196b or the vector. **d** Real-time migration of HCT 116 cells transfected with pLV-miR-196b or the vector. The delta cell index indicates electrical impedance. **e** Transwell invasion assays were used to assessed the effects of miR-196b on the HCT 116 cell invasion ability. **f**–**g** The effects of miR-196b mimics or miR-196b inhibitors on the migration of SW480 cells with METTL3 knockdown or overexpression were determined by Transwell migration assays. The data are presented as the mean ± SD (*P < 0.05, **P < 0.01, ***P < 0.001; Student’s *t* test)

To further investigate the pathological and prognostic significance of miR-196b expression in CRC patients, the miR-196b levels in TMA slides containing 283 CRC samples were quantified using ISH. Positive tissue staining was indicated by blue‒violet color (Fig. 6a). Our results showed that miR-196b was upregulated in patients with lymph node metastasis compared with those without lymph node metastasis (Fig. 6b), and miR-196b expression was correlated with TNM stage (*P* = 0.050), lymph node metastasis (pN) (*P* = 0.003) and recurrence (*P* = 0.028) (Table 1). To determine the role of miR-196b in CRC cell metastasis, CRC cells were stably infected with the miR-196b or scramble control lentiviral vector. Increased miR-196b expression in the cells following infection was confirmed by qRT-PCR (Fig. 6c and Fig. S5a). Real-time cell migration assays showed that overexpression of miR-196b significantly promoted CRC cells migration (Fig. 6d and Fig. S5b). Transwell invasion assays showed that ectopic miR-196b expression significantly increased the invasion of CRC cells (Fig. 6e and Fig. S5c). Taken together, these findings indicated that miR-196b increases migration and invasion of CRC cells.

**Table 1:** Characteristics of miR-196b expression based on clinical parameters from 283 CRC patients

Factors	Number of cases	miR-196b expression		*P* value
		Low (%)	High (%)	
Age				
< 60	133	59 (44.4)	74 (55.6)	0.084
≥ 60	150	82 (54.7)	68 (45.3)	
Gender				
Female	125	60 (48.0)	65 (52.0)	0.585
Male	158	81 (51.3)	77 (48.7)	
Location				
Colon	131	65 (49.6)	66 (50.4)	0.950
Rectal	140	70 (50.0)	70 (50.0)	
Tumor size				
< 5 cm	138	71 (51.4)	67 (48.6)	0.475
> 5 cm	142	67 (47.2)	75 (52.8)	
TNM stage				
I + II	161	88 (54.7)	73 (45.3)	0.050*
III + IV	117	50 (42.7)	67 (51.3)	
pT				
T1 + T2	58	34 (58.6)	24 (41.4)	0.110
T3 + T4	222	104 (46.8)	118 (53.2)	
pN				
N0	172	98 (57.0)	74 (43.0)	0.003*
N1 + N2	104	40 (38.5)	64 (61.5)	
pM				
M0	260	128 (49.2)	132 (50.8)	0.503
M1	23	13 (56.5)	10 (43.5)	
Differentiation				
High	24	12 (50.0)	12 (50.0)	0.881
Middle	228	111 (48.7)	117 (51.3)	
Low	26	14 (53.8)	12 (46.2)	
Recurrence				
Yes	11	9 (81.8)	2 (18.2)	0.028*
No	265	127 (47.9)	138 (52.1)	

*P < 0.05, Chi-square test

Next, we transfected miR-196b mimics into METTL3 knockdown cells, and found that the miR-196b mimics could partly increase CRC cell migration inhibited by METTL3 knockdown (Fig. 6f). Accordingly, miR-196b inhibitors partially decreased the increase in cell migration induced by METTL3 overexpression (Fig. 6g).

## Discussion

Recent studies have shown the important role of m6A modification in regulating RNA metabolism and various biological processes (Chen et al. 2020b; Roundtree et al. 2017; Kasowitz et al. 2018; Du et al. 2016; Wang et al. 2014, 2015; Weng et al. 2018; Xu et al. 2017; Zhong et al. 2018). In the present study, we show that METTL3 promotes CRC migration by accelerating the maturation of pri-miR-196b in an m6A-dependent manner.

We found that METTL3 expression is significantly upregulated in CRC tissues and promotes CRC cell migration and invasion. These results are consistent with those reported by other studies (Li et al. 2019; Zhou et al. 2021a; Chen et al. 2021a; Pan et al. 2022). Chen et al. identified METTL3 as the most essential m6A regulatory enzyme that is overexpressed in CRC (Chen et al. 2021a). Pan et al. reported that m6A and METTL3 levels were substantially elevated in CRC tissues, and patients with CRC with high m6A or METTL3 levels exhibited shorter overall survival (Pan et al. 2022). In CRC, METTL3 has been reported to regulate metastasis by m6A-dependent posttranscriptional modification of SOX2, HK2, GLUT1, YPEL5 and CRB3 (Li et al. 2019; Zhou et al. 2021a; Shen et al. 2020; Pan et al. 2022). (Li et al. 2019; Zhou et al. 2021a) However, METTL3 has also been reported as a tumor suppressor in CRC. Deng et al. found that METTL3 is significantly associated with longer survival time and suppresses CRC cell proliferation, migration and invasion through p38/ERK pathways (Deng et al. 2019). These contradictory conclusions reached in previous studies must be related to the use of different modification sites, various m6A-binding readers and multiple downstream targets.

miRNAs are short noncoding RNAs that play critical roles in diverse biological processes, and their aberrant expression has been associated with numerous human diseases (Andriani et al. 2018; Meng et al. 2013; Wang et al. 2017; El Bezawy et al. 2017; Kim et al. 2014; Ling et al. 2016). The m6A methyltransferase, m6A-binding proteins and demethylase have been reported to affect miRNA expression levels (Berulava et al. 2015; Alarcon et al. 2015a, b; Han et al. 2019; Wang et al. 2019; Bi et al. 2021; Peng et al. 2019). METTL3 was found to methylate pri-miRNAs, which marked them for recognition and processing by the microprocessor complex protein DGCR8 (Alarcon et al. 2015b). To investigate whether METTL3 regulates CRC metastasis through miRNAs, we measured the global impact of METTL3 depletion on miRNA levels. The miRNA-seq results showed that the expression levels of miR-21, miR-196b and miR-1246 were most significantly downregulated after METLL3 knockdown. Previous studies have reported that m6A modification-dependent pri-miRNA processing is essential for the maturation of miR-21-5p (Liu et al. 2021; Wang et al. 2021) and miR-1246-5p (Huang et al. 2021; Peng et al. 2019), but its role in the maturation of miR-196b has not yet been investigated.

To investigate whether METTL3 regulates miR-196b expression through m6A modification, we first analyzed the pri-miR-196b sequence using SRAMP. The results of bioinformatic prediction showed several RRACH motifs in the pri-miR-196b sequence. We also found that the RRACH motif is not present in the pre-miR-196b regions but is located in single-stranded RNA flanking the pri-miRNA hairpin. This finding is consistent with the results of the work of Alarcon et al., who used the Finding Informative Regulatory Elements algorithm to identify overrepresentation of the GGAC motif in pri-miRNA sequences; that study demonstrated that the GGAC motif is not enriched in pre-miRNA sequences (Alarcon et al. 2015b). A previous study showed that METTL3 methylates the single-stranded RNA flanking the pri-miRNA hairpin, marking these structures for recognition and processing by DGCR8, and that METTL3 depletion reduced the binding of DGCR8 to pri-miRNAs and resulted in a reduction in mature miRNAs and the concomitant accumulation of unprocessed pri-miRNAs (Alarcon et al. 2015b). Therefore, we hypothesized that METTL3 targets pri-miR-196b for m6A modification, and promotes pri-miR-196b processing. To assess our hypothesis, we designed PCR primers to amplify the putative m6A region of pri-miR-196b. The qRT-PCR results showed that METTL3 knockdown significantly decreased the expression of miR-196b but increased the expression of pri-miR-196b in CRC cells, while the overexpression of METTL3 increased the expression of miR-196b but reduced the expression of pri-miR-196b in CRC cells. Furthermore, our results revealed that METTL3 interacts with the m6A methylation region of pri-miR-196b, and the m6A modification level of pri-miR-196b was significantly reduced following the silencing of METTL3. Taken together, our results showed that METTL3 regulates miR-196b expression through m6A modification.

An increasing number of studies have shown that miR-196b has dual functions as both a tumor promoter (Yu et al. 2016; Liao et al. 2012) and a suppressor of cancer progression (How et al. 2013; Tellez et al. 2016). In the present study, we confirmed that miR-196b is upregulated in CRC and that overexpression of miR-196b promotes CRC cell migration and invasion, which is consistent with previous reports of miR-196b as an onco-miRNA in CRC (Stiegelbauer et al. 2017). Furthermore, our in vitro gain-of-function and rescue experiments substantiated that METTL3 promotes CRC migration invasion, at least partially, in an miR-196b-dependent manner.

## Conclusions

Taken together, our results show that METTL3 expression is significantly increased in CRC and that METTL3 promotes CRC cell migration and invasion. Moreover, we first demonstrate that METTL3 promotes CRC metastasis by accelerating pri-miR-196b maturation in an m6A-dependent manner; this finding may provide a potential therapeutic target for antimetastatic strategies against CRC.

## Supplementary Information

Below is the link to the electronic supplementary material.

Supplementary file1 (PDF 580 KB)

Supplementary file2 (XLSX 52 KB)
