# Supplementary material for: METTL3 promotes colorectal cancer metastasis by promoting the maturation of pri-microRNA-196b
Source: J Cancer Res Clin Oncol. 2022 Nov 8;149(8):5095–108. doi: 10.1007/s00432-022-04429-9 (PMC10349789; doi:10.1007/s00432-022-04429-9)
Supplement: Supplementary file 1 — Supplementary file1 (PDF 580 KB) [file 432_2022_4429_MOESM1_ESM.pdf]

METTL3 promotes colorectal cancer metastasis by promoting the maturation of pri-microRNA-196b

Lanlan Huang[1,2,3], Danlu Liang[2], Yu Zhang[2], Xiaoting Chen[2], Junxiong Chen[3], Chuangyu Wen[3], Huanliang Liu[3,4], Xiaorong Yang[1], Xiangling Yang[3*], Shaoqiang Lin[2*]

[1] Department of Clinical Laboratory, the First Affiliated Hospital of Guangdong Pharmaceutical University, Guangzhou, China.

[2] School of Clinical Medicine, Guangdong Pharmaceutical University, Guangzhou, China.

[3] Guangdong Provincial Key Laboratory of Colorectal and Pelvic Floor Diseases, Guangdong Institute of Gastroenterology, the Sixth Affiliated Hospital, Sun Yat-sen University, Guangzhou, China.

[4] Department of Clinical Laboratory, the Sixth Affiliated Hospital, Sun Yat-sen University, Guangzhou, China.

[*]Correspondence: sqlin123@163.com; yangxl28@mail.sysu.edu.cn

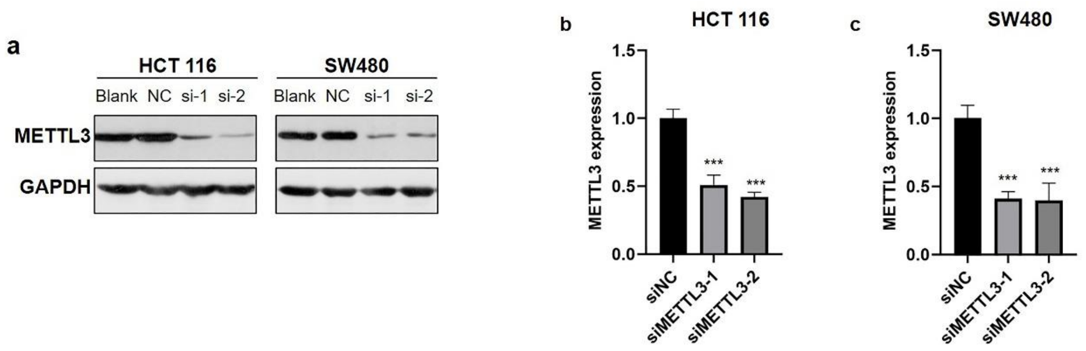

Figure S1 The expression levels of METTL3 in CRC cells. **a** The expression levels of METTL3 in HCT 116 and SW480 cell lines transfected with siRNA determined by western blot. **b** The expression levels of METTL3 in HCT 116 and SW480 cell lines transfected with siRNA determined by qRT–PCR.

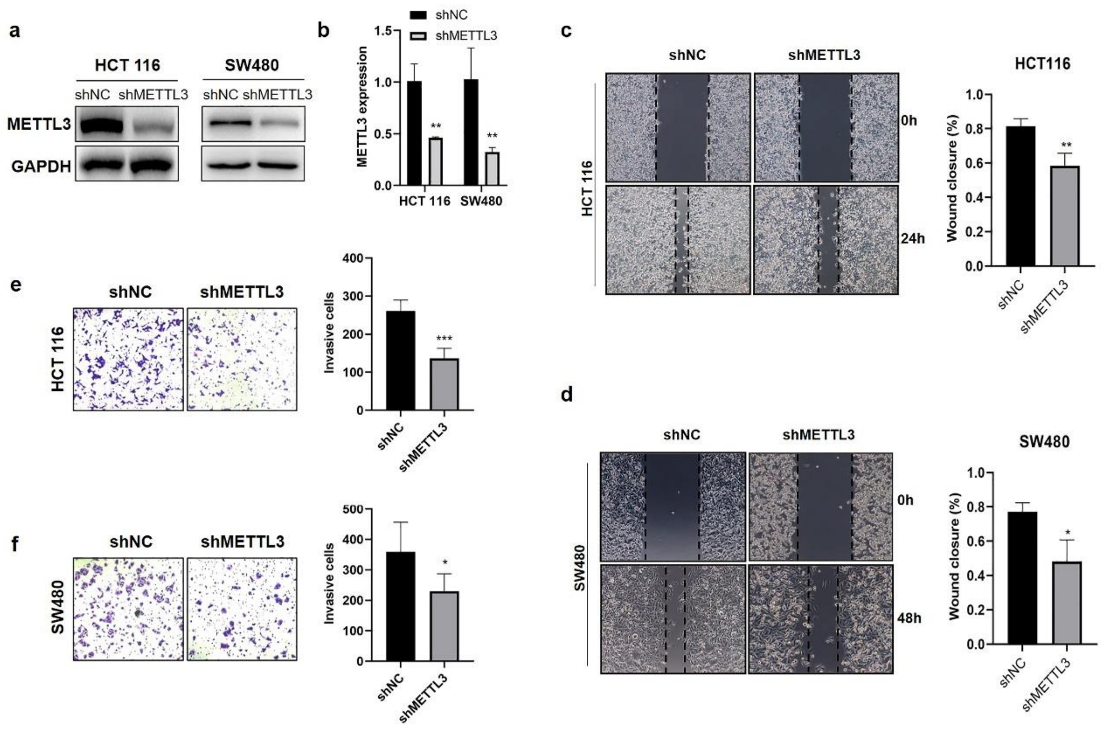

Figure S2 METTL3 knockdown inhibited CRC cell migration and invasion *in vitro*. **a-b** Western blot and qRT–PCR analysis of the expression of METTL3 in CRC cells infected with shNC or shMETTL3. **c-d** Wound healing assays were performed to investigate the effects of downregulation of METTL3 on the migration ability of CRC cells. **e-f** Transwell invasion assays were performed to estimate the effects of downregulation of METTL3 expression on CRC cell invasion (The quantitative data are presented in the histograms and were assessed with a two-tailed unpaired Student's *t* test. Data are presented as the mean ± SD. *$P < 0.05$, **$P < 0.01$, ***$P < 0.001$).

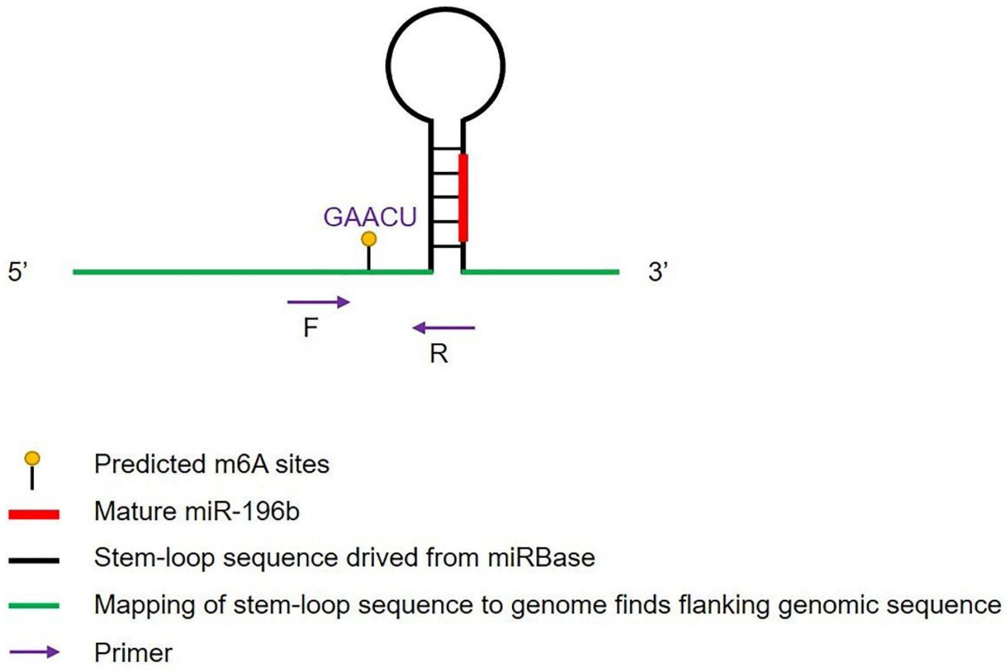

Figure S3 Amplification of the pri-miR-196b regions.

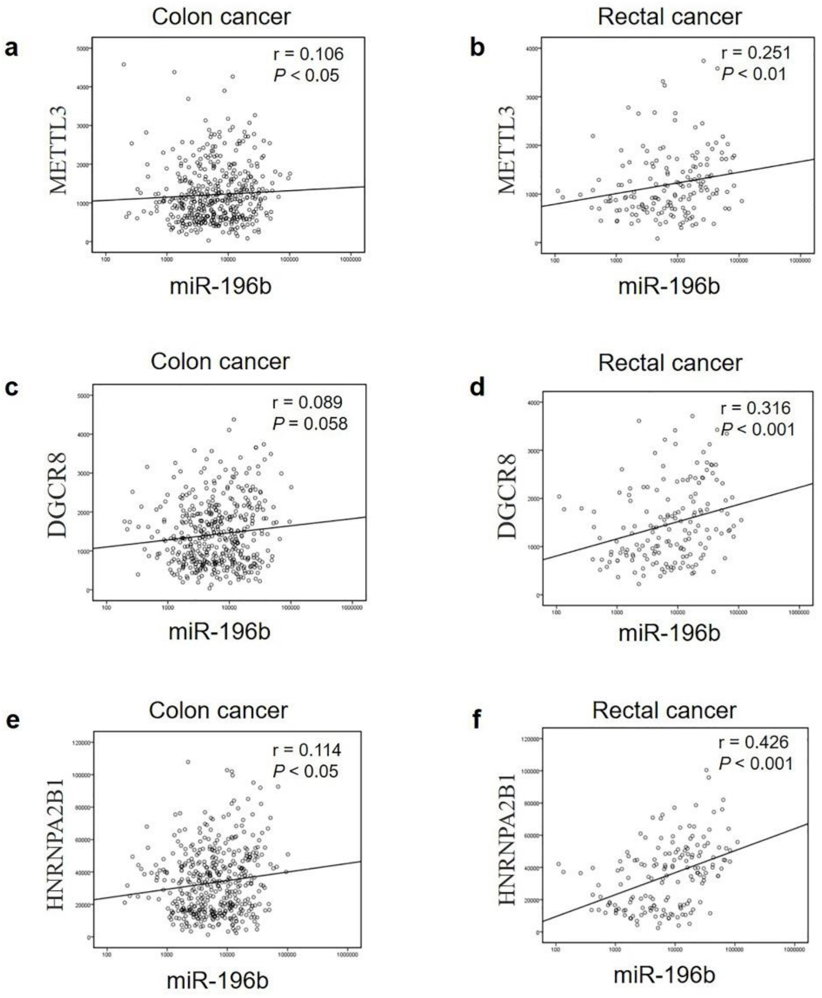

Figure S4 miR-196b expression positively correlated with METTL3, DGCR8 and HNRNPA2B1 in CRC tissues. **a-b** Correlation analysis of miR-196b and METTL3 mRNA expression in human CRC tissues. **c-d** TCGA database illustrated the correlation between miR-196b and DGCR8 expression. **e-f** miR-196b expression showed positively correlated with HNRNPA2B1 mRNA levels in CRC tissues.

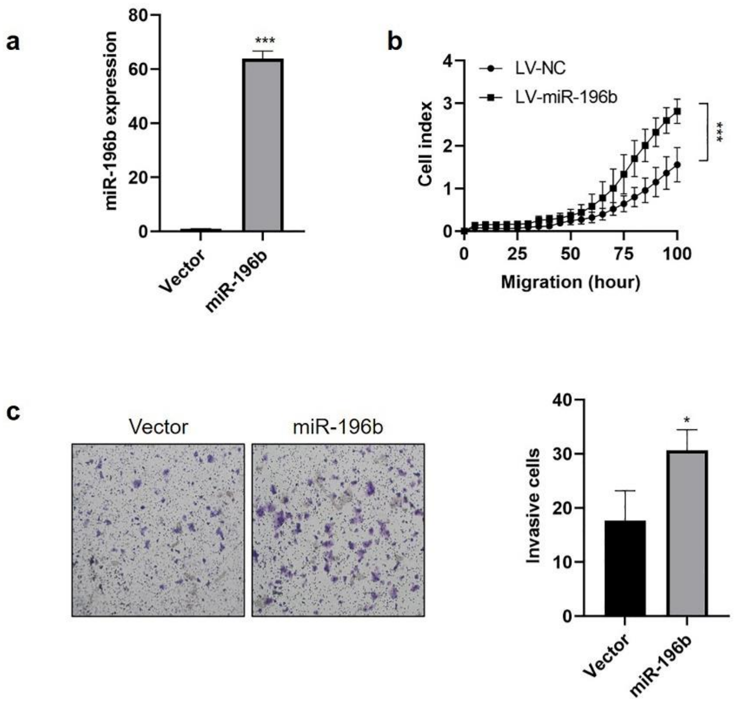

Figure S5 Overexpression of miR-196b promotes Caco2 cell migration and invasion. **a** qRT–PCR analysis of miR-196b expression in Caco2 cells infected with pLV-miR-196b or the vector. **b** Real-time migration of Caco2 cells transfected with pLV-miR-196b or the vector. The delta cell index indicates electrical impedance measurements. **c** Transwell invasion assays were used to estimate the effects of miR-196b on the Caco2 cell invasion ability.
